# Demographic, clinical and aetiological characteristics of patients with hepatocellular carcinoma followed between 2012 and 2017 at University Hospital Joseph Raseta Befelatanana, Antananarivo, Madagascar

**DOI:** 10.3332/ecancer.2022.1466

**Published:** 2022-11-04

**Authors:** Andry Lalaina Rinà Rakotozafindrabe, Chantelli Iamblaudiot Razafindrazoto, Domoina Harivonjy Hasina Laingonirina, Tojonokoloina Benjamin Ralideramanambina, Behoavy Mahafaly Ralaizanaka, Sonny Maherison, Jolivet Auguste Rakotomalala, Nitah Harivony Randriamifidy, Anjaramalala Sitraka Rasolonjatovo, Tovo Harimanana Rabenjanahary, Soloniaina Hélio Razafimahefa, Rado Manitrala Ramanampamonjy

**Affiliations:** 1 Department of Gastroenterology, University Hospital Joseph Raseta Befelatanana, Antananarivo 101, Madagascar; 2 Department of Hepato-Gastroenterology, University Hospital Andrainjato, BP1487 Ambatoharanana, Fianarantsoa 301, Madagascar; 3 Department of Hepato-Gastroenterology, University Hospital Mahavoky Atsimo, Mahajanga 401, Madagascar

**Keywords:** hepatocellular carcinoma, risk factors, hepatitis B virus, Madagascar

## Abstract

**Purpose:**

The aim of this study was to describe the demographic, clinical and aetiological characteristics of hepatocellular carcinoma (HCC) in a Malagasy population sample in view to defining an appropriate control program.

**Methods:**

This was a retrospective, descriptive study conducted in the Gastroenterology Department, Joseph Raseta Befelatanana University Hospital, Antananarivo, over a period of 6 years (January 2012 to December 2017).

**Results:**

A total of 42 patients were selected, 29 of whom were men (69.05%) and 13 women (30.95%) (sex ratio: 2.2). The mean age was 56.6 years with extremes of 21 and 82 years. Subjects aged 60–69 years were most affected (35.71%). Abdominal pain was the main revealing symptom (38.10%). The main aetiological factors were: hepatitis B virus (HBV) (42.86%), hepatitis C virus (19.05%) and chronic alcoholism (23.81%). All patients were cirrhotic, of which 23 patients (54.76%) had Child–Pugh B class and 15 (35.71%) Child–Pugh C. Twenty-six patients (61.90%) had α-foetoprotein level plus 500 ng/mL. Six patients (14.29%) had portal thrombosis at diagnosis. Twenty patients (47.62%) had advanced HCC (Barcelona Clinic Liver Cancer C (BCLC C)) and 21 (50%) had end-stage HCC (BCLC D). Management was palliative in 41/42 patients. The in-hospital death rate was 23.81%.

**Conclusion:**

HCC are diagnosed at advanced stage in this study. The prognosis is poor for most patients. HBV infection is the main risk factor. An effort should be made for early diagnosis and prevention.

## Background

Hepatocellular carcinoma (HCC) is the most common primary malignancy of the liver. In most cases, it occurs in patients with chronic liver disease, most often in the cirrhosis stage [1, 2]. Primary liver cancer is the sixth most commonly diagnosed cancer and the third leading cause of cancer death world-wide in 2020, with approximately 906,000 new cases and 830,000 deaths. Rates of both incidence and mortality are 2–3 times higher among men than among women in most regions, and liver cancer ranks fifth in terms of global incidence and second in terms of mortality for men (Globocan 2020 data) [3]. Published incidences of HCC in the Black population of sub-Saharan Africa underestimate the true incidence of the tumour because of the many instances in which HCC is either not definitively diagnosed or is not recorded in a cancer registry. Forty-six thousand new cases of HCC have been recorded to be diagnosed in sub-Saharan Africa each year, and age-standardised incidences of the tumour as high as 41.2/100,000 persons/year have been documented. The highest incidence of HCC has been recorded in Mozambique [4, 5]. Furthermore, in Africa and Southeast Asia, where hepatitis B virus (HBV) infection is acquired early in life and coincides with other oncogenic agents (e.g. aflatoxin), HCC may develop more frequently in a non-cirrhotic liver [6]. A recent Malagasy study (2019) reported that HCC was the second most common gastrointestinal cancer after colorectal cancer [7]. The curative means of HCC are complex, expensive and depend on local resources [8, 9]. This management is not yet within the reach of all Africans, as in Madagascar [8, 10, 11]. In order to better understand this problem, it is essential to have a good knowledge of the aspect of HCC in each country. Thus, the objective of this study was to describe the demographic, clinical and aetiological characteristics of HCC at the University Hospital Joseph Raseta Befelatanana, Antananarivo, Madagascar.

## Materials and methods

### Study design and study population

This was a retrospective, descriptive study conducted in the Gastroenterology Department, University Hospital Joseph Raseta Befelatanana, Antananarivo, over a period of 6 years from January 2012 to December 2017. This Hospital is a public tertiary hospital treating internal medicine pathologies. Gastroenterology department provides care for all patients with acute and chronic diseases of the liver, pancreas and digestive tract, including screening for digestive cancers. The department has 27 conventional hospitalisation beds and 2 functional units including a general hepato-gastroenterology unit and an endoscopy unit. Patients are generally recruited via the emergency services. Institutional ethics approval was not required for this study, nor was written informed consent required. Permission from the hospital’s scientific director was obtained to conduct the study. All records of patients hospitalised, during the study period, with one or more liver nodules were collected. HCC remains a major exception in oncology, as a conclusive diagnosis can be obtained in a significant proportion of patients by imaging techniques, without the need for histologic confirmation [12]. All the files with a diagnosis of HCC were included. The following were excluded from the study: hepatic nodules smaller than 1 cm, without α-foetoprotein (AFP) elevations and not having been biopsied; multiple hepatic nodules with an AFP level < 400 ng/mL, with concomitant presence of another tumour with hepatic metastatic potential; large hepatic nodules, without typical character of HCC and not having an AFP > 400 ng/mL. Sociodemographic data (age, gender, activity sectors), clinical data (comorbidities, reasons for consultation, aetiological factors, World Health Organization (WHO) performance status index (PSI)), biological data (liver tests and AFP levels), tumour characteristics (number, size, portal or distant invasion) and patient outcome were collected.

### Methodology

The diagnosis of HCC was made on the basis of the following clinico-biological and radiological arguments: (1) Presence of one or more nodules on a healthy or cirrhotic liver whose character is hyper-vascularised at the arterial time associated with a washout at the portal time, (2) an elevation of the AFP level more than 400 ng/mL, which was not mandatory if the radiological character was typical. The diagnosis of cirrhosis was based on clinical (signs of hepatocellular insufficiency, signs of portal hypertension, liver with sharp lower border), biological (biological signs of hepatocellular insufficiency and/or F4 fibrosis on fibrotest) and morphological (ultrasound and endoscopic signs of portal hypertension, liver features on abdominal ultrasound) arguments.

The viral origin of the HCC was retained in view of the positivity of the viral markers (HBV surface antigen and anti-hepatitis C virus (anti-HCV) antibody). The alcoholic aetiology was retained on the basis of an alcoholic consumption of more than 20 g/days in women and 40 g/days in men for at least 10 years, and without other aetiologies found. Non-alcoholic steatohepatitis was evoked in case of metabolic syndrome in a non-alcoholic patient, without other causes found. The diagnosis of cryptogenic HCC was a diagnosis of elimination and was based on the following: (1) no serologic or clinical evidence of HBV or HCV infection; (2) alcohol consumption < 20 g/day in men (<10 g/day in women) and (3) no evidence of other causes of chronic liver disease.

### Data collection and statistical analysis

Data were collected from patient registers and medical records and entered using Microsoft Excel. Data were analysed using EPI-info version 7.2 software. Quantitative variables were expressed as mean standard deviation or median and qualitative variables as numbers and percentages. The anonymity of the files was respected.

## Results

### Epidemiological characteristics and aetiological factors of HCC

During our study period, 115 patients were hospitalised at University Hospital Joseph Raseta Befelatanana for a malignant liver tumour, of which 42 patients were hospitalised for HCC, a frequency of 36.52% of liver tumours. Our study population was made up of 29 men (69.05%) and 13 women (30.95%), giving a sex ratio of 2.2. The average age of our patients was 56.6 ± 15.6 years with a range of 21-82 years. Patients aged 60–69 years and workers in the primary and secondary sectors were the most affected with a respective rate of 35.71% (*n* = 15), 38.10% (*n* = 16) and 45.24% (*n* = 19). Twenty-nine patients (69.05%) were smokers. Abdominal pain (38.10%) and decompensation of cirrhosis (42.85%) were the main revealing symptoms of the disease. All our patients were cirrhotic, including 4 (9.52%) Child–Pugh A class, 23 (54.76%) Child–Pugh B and 15 (35.71%) Child–Pugh C. The aetiological factors found were HBV (42.86%), HCV (19.05%), HBV/HCV co-infection (7.14%) and alcohol (23.81%). The sociodemographic and clinico-biological characteristics are represented in Tables 1 and 2.

### Tumour characteristics, type of management and in-hospital outcome

**Figure 1.**
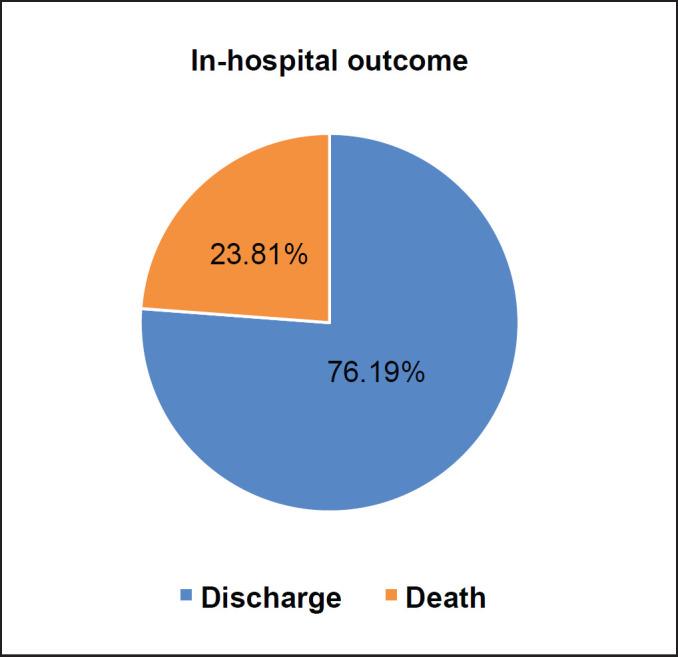
In-hospital outcome of our study population.

The majority of patients had a WHO PSI of 1–2 (42.86%) and PSI > 2 (45.24%) at diagnosis. Twenty-six patients (61.90%) had AFP > 500 ng/mL, 8 (19.05%) 200–500 ng/mL and 8 (19.05%) < 200 ng/mL. The liver nodule was single in 9 patients (21.43%) and multiple (≥2) in 33 patients (78.57%). More than half of the patients had a localised tumour (54.76%) and less than 5 cm in diameter (76.19%). The disease was metastatic (locoregional and distant) in 19 patients (45.24%). Six patients (14.29%) had portal thrombosis at the time of diagnosis. According to the Barcelona Clinic Liver Cancer (BCLC) classification, the majority of patients had advanced (BCLC C, 47.62%) and end-stage (BCLC D, 50%) HCC. Management was palliative in 41/42 patients (97.62%). Ten patients (23.81%) had died during hospitalisation (Figure 1). Complications of cirrhosis occupied all causes of death. The characteristics of the tumour and the type of management are detailed in Table 2.

## Discussion

This work was a 6-year retrospective analysis of HCC cases in Madagascar. Although our sample size was not representative of the Malagasy population, this study was essential because it allowed us to make an epidemiological, clinical, radiological and aetiological description of HCCs in a Malagasy population sample.

This study had some limitations. Its retrospective character did not allow us to collect other information such as the results of the liver biopsy, not allowing us to know the histology, the molecular and genetic aspect of each tumour. Small nodules that did not have typical features on imaging were dismissed out of hand without histology and underestimating the prevalence of HCC. In this study, the AFP assay was still a cornerstone of the diagnosis because imaging could not go beyond a CT scan because liver MRI was not yet available.

HCC is a major public health problem worldwide [13, 8]. HCC is a common malignant liver tumour among liver tumours. It requires special attention in daily practice. It is the third most common digestive cancer in Senegal [14], and a frequent reason for hospitalisation in the hepato-gastroenterology department in Burkina Faso (50%–70% of hospitalised patients) [8, 10]. A 2017 Malagasy data (oncology service of the military hospital of Antananarivo) on cancer epidemiology reported that the first common neoplasia was colorectal cancers (12.15%) followed by haematological malignancies of lymphoid tissues (8.28%) and pulmonary cancers (5.52%). However, cancer remains the first neoplasia in women. According to this study, primary liver cancer (1.10%) remains the 20th cancer in Madagascar. These data on primary liver tumours remain underestimated [15]. A study conducted in Fianarantsoa Madagascar in 2019 by Ranaivomanana *et al* [7] reported that HCC was the second most common gastrointestinal cancer after colorectal cancer.

Its incidence has been increasing over the last 20 years in developed countries. Several factors explain this growth: increase in HCV-related HCC cases, improved diagnostic tools and better management of other complications of cirrhosis [16].

Different from Western countries, HCC affects the young population in Sub-Saharan Africa [5, 17]. In the present study, HCC affected patients older than 50 years with a mean age of 56.6 ± 15.6 years and a peak frequency between 60 and 69 years. They were relatively young despite the discrepancy found compared to those in other neighbouring countries [8, 9, 11, 14, 17, 18–24]. Prolonged exposure to HBV and HCV would explain the occurrence of HCC in these patients. These factors are major in sub-Saharan Africa. In addition, these populations may also be exposed to cofactors such as aflatoxin B1 which is a potent dietary carcinogen, but this remains to be verified for the Malagasy population [13, 20, 25].

A clear male predominance was observed in our series, consistent with all the data in the Western [1, 13, 26], Asian [27–32] and African [8–11, 14, 17, 18–24] literature. Risk factors for HCC such as HBV, HCV, alcoholism and smoking would be more frequent in men than in women [1, 2, 20]. In addition, hormonal factors such as oestrogen have been described as a protective factor in women [20, 33].

In Madagascar, the diagnosis of HCC was based on non-invasive criteria (imaging and AFP level). AFP plays an important role in the diagnosis of HCC in Madagascar. AFP was positive in all our patients, and more than half (61.90%) of the patients had a level higher than 500 ng/mL. According to the literature, the specificity of AFP was 100% at a level of 500 ng/mL [34]. The contribution of AFP as a diagnostic test remains limited due to its low sensitivity (39%–65%), despite its specificity of 76%–94% [35]. Nevertheless, it is well known that AFP may play an important prognostic role in the follow-up of these patients, as high AFP levels may signal more aggressive, multifocal tumours associated with venous portal thrombosis and/or metastasis [36].

HBV remains the leading cause of HCC worldwide (55%), where the majority of cases occur in Africa (70%–80% of liver cancer patients carry HBV or its markers) [10, 11]. In the present study, HBV was the main cause of HCC in our patients. Our results confirmed the finding of a previous Malagasy data (1996) reporting that HBV was the main cause of HCC [37]. HCV and alcoholism were the other two most common risk factors. These data were in line with the African [8–11,14, 17, 18–25] and Asian [27–32] literature. On the other hand, HCV was the leading cause of HCC in a Nigerian [21] and Japanese [29, 30] study, suggesting an evolution of the aetiological profile of HCC in these regions. In Asia, a decline in the incidence of HBV-associated HCC had been noted over the last 12 years [29]. In a prospective Asian cohort including 3,349 patients with HCC, the authors had found several major chronological changes between 2004 and 2015. HCC patients with hepatitis B or C gradually decreased between 2004–2007 and 2012–2015 [29, 31]. The launch of the mass vaccination programme against hepatitis B and specific treatments for HBV and HCV contributed to this decrease in the prevalence of HCC related to viral hepatitis in Asian countries [29–31]. In our series, 69.05% of patients were smokers. However, the carcinogenic role of tobacco remains controversial as few studies have shown an association of tobacco with HCC [38]. Aflatoxin is an additional risk factor for HCC in Africa due to regular consumption of peanuts and corn [20, 25, 39]. Aflatoxin is responsible for 5%–25% of liver cancer cases [25]. An Egyptian study found a positive aflatoxin B1 test in 17% of HCC patients [40]. This test is not yet available in Madagascar.

In our series, the majority of patients were BCLC D/C, symptomatic and had an PSI > 2, signifying a very advanced stage of the disease. The same findings had been made in Nigeria [21], Cameroon [23], Congo [19], Burkina Faso [24] and in the rest of Sub-Saharan African countries [8–11, 17, 18, 22]. Whereas in Western countries, HCC is discovered very early in the majority of cases [26]. This suggests that an effort is needed for the early diagnosis of HCC in the Malagasy population. The development of a hospital and population-based cancer registry remains highly recommended in Madagascar to improve the Ministry of Health’s strategy in view of the demographic, clinical and aetiological characteristics of patients with different neoplasia’s, including HCC.

As expected, one patient had benefited from specific HCC treatment in our series. Our data confirmed the results of a recent African multicentre study on HCC. The study reported that specific management and curative treatment of liver cancer was only possible in 3% and less than 1% of patients, respectively [8, 10, 17]. Adequate management of HCC is difficult in sub-Saharan Africa, particularly in Madagascar where specific treatments are not available [8, 10, 11]. Therefore, the management of HCC should focus on the prevention of risk factors, especially viral hepatitis and alcoholism. Monitoring of cirrhosis by ultrasound every 6 months should be systematic for practitioners.

## Conclusion

HCC has a high prevalence among liver tumours in this Malagasy population sample. The risk factors are common to those described from sub-Saharan Africa, notably the abundance of HBV-related HCC. Efforts are still needed in terms of diagnosis and specific care. Promotion of hepatitis B vaccination and surveillance of cirrhotic patients for early detection of HCC could improve the management of HCC in Madagascar.

## Abbreviations

AFP, α-foetoprotein; BCLC, Barcelona Clinic Liver Cancer; HCC, Hepatocellular carcinoma; HBV, Hepatitis B virus; HCV, Hepatitis C virus; PSI, Performance status index; WHO, World Health Organization.

## Conflicts of interest

No conflicts of interest to declare.

## Funding statement

This research was not supported by any specific grants from public, commercial or nonprofit funding agencies.

## Authors’ contributions

All authors adhere to the International Committee of Medical Journal Editors (ICMJE) definition of authorship and they have complete access to the study data that support the publication. CIR: writing draft, reviewing and editing, software, data curation, methodology and formal analysis; ALRR, DHHL, TBR and RMR: Conceptualisation, data curation, validation, reviewing and editing, software, methodology and formal analysis; BMR, SM, JAR, NHR, ASR, THR and SHR: methodology, visualisation, validation, reviewing and editing the draft. All authors read and approved the final manuscript.

## Figures and Tables

**Table 1.** Socio-demographic characteristics of HCC patients at University Hospital Joseph Raseta Befelatanana (*n* = 42).

Variables	*N* (%)
Gender	
Male	29 (69.05)
Female	13 (30.95)
Age range (years)	
	1 (2.38)
30–39	4 (9.52)
40–49	7 (16.66)
50–59	7 (16.66)
60–69	15 (35.71)
70–79	7 (16.66)
≥80	1 (2.38)
Sectors of activity	
Primary sector	16 (38.10)
Secondary sector	19 (45.24)
Tertiary sector	3 (7.14)
Others	4 (9.52)

**Table 2.** Clinico-biological, radiological and therapeutic characteristics of HCC patients at HUJRB (*n* = 42).

Variables	*N* (%)
Smoking, Yes/no	29 (69.05)/13 (30.95)
Revealing symptomatology’s	
Abdominal pain	16 (38.10)
Alteration of the general sate	1 (2.38)
Decompensation of cirrhosis	18 (42.85)
Jaundice	5 (11.90)
Abdominal mass	2 (4.76)
PSI, 0/1–2/>2	5 (11.90)/18 (42.86)/19 (45.24)
AFP level (ng/mL)	
<200	8 (19.05)
200–500	8 (19.05)
>500	26 (61.90)
Total bilirubin (μmol/L), <20/20–30/>30	4 (9.52)/15 (35.71)/23 (54.76)
ALT (U/L), ≤45/>45	5 (11.90)/37 (88.10)
Cirrhosis, Yes/no	42 (100)/0 (0)
Child–Pugh class, A/B/C	4 (9.52)/23 (54.76)/15 (35.71)
Aetiological factors	
HBV	18 (42.86)
HCV	8 (19.05)
HBV/HCV	3 (7.14)
Alcohol	10 (23.81)
Cryptogenic	3 (7.14)
Nodule size (cm), <3/3–5/>5	18 (42.86)/14 (33.33)/10 (23.81)
Number of nodule, Single/multiple (≥2)	9 (21.43)/33 (78.57)
Stage of tumour	
Localised	23 (54.76)
Locoregional invasion	16 (38.10)
Distant metastasis	3 (7.14)
Portal thrombosis, Yes/no	6 (14.29)/36 (85.71)
BCLC stages	
A	1 (2.38)
B	0 (0)
C	20 (47.62)
D	21 (50)
Treatments	
Curative	1 (2.38)
Palliative	41 (97.62)

AFP, α-foetoprotein; ALT, Alanine aminotransferase; HBV, Hepatitis B virus infection; HCV, Hepatitis C virus infection; BCLC, Barcelona Clinic Liver Cancer

## References

[1] Blanc JF (2017). Carcinome hépatocellulaire: nouveautés épidémiologiques et démarche diagnostique. Oncologie.

[2] Lambert R (2009). Épidémiologie du carcinome hépatocellulaire (CHC) dans le monde. Cancéro Dig.

[3] Sung H, Ferlay J, Siegel RL (2021). Global cancer statistics 2020: estimations GLOBOCAN de l’incidence et de la mortalité dans le monde pour 36 cancers dans 185 pays. CA Cancer J Clin.

[4] Michael CK (2013). Epidemiology of carcinome hépatocellulaire en Afrique sub-saharienne. Ann Hepatol.

[5] Prates MD, Torres FO (1965). Une enquête sur le cancer à Lourenco Marques, Afrique orientale portugaise. J Natl Cancer Inst.

[6] Bruix J, Sherman M, Llovet JM (2001). Clinical management of hepatocellular carcinoma. Conclusions of the Barcelona-2000 EASL conference Eur Association for the Study of the Liver. J Hepatol.

[7] Ranaivomanana M, Rafaralahy T, Razafimahefa J (2019). Epidemiology of gastrointestinal cancers in the hospital of Tambohobe Fianarantsoa, Madagascar. Int J Res Med Sci.

[8] Yang JD, Mohamed EA, Aziz AOA (2017). Characteristics, management, and outcomes of patients with hepatocellular carcinoma in Africa: a multicountry observational study from the Africa liver cancer consortium. Lancet Gastroenterol Hepatol.

[9] Umoh NJ, Lesi OA, Mendy M (2011). Aetiological differences in demographical, clinical and pathological characteristics of hepatocellular carcinoma in the Gambia. Liver Int.

[10] Sombié R (2017). Un drame africain: à quand le fin carcinome hépatocellulaire. J Afr Hépatol Gastrentérol.

[11] Ntagirabiri R, Munezero B, Kaze H (2015). Incidence du carcinome hépatocellulaire lors de l’infection chronique par le virus de l’hépatite B. Pan Afr Med J.

[12] Forner A, Da Fonseca LG, Díaz-González Á (2019). Controversies in the management of hepatocellular carcinoma. JHEP Rep.

[13] Fares N, Péron JM (2016). Le carcinome hépatocellulaire au cours de l’infection virale B. Hépato Gastro.

[14] Ba PA, Wade TMM, Diao ML (2021). Gastrointestinal cancer in young adults: report of 77 cases at Aristide le Dantec teaching hospital of Dakar. Clin Oncol.

[15] Hasiniatsy NRE, Ramahandrisoa AVN, Refeno V (2017). Épidémiologie des cancers pris en charge en oncologie médicale à l’hôpital militaire d’Antananarivo, Madagascar. Bull Cancer.

[16] Trinchet JC (2009). Hepatocellular carcinoma: increasing incidence and optimized management. Gastroenterol Clin Biol.

[17] Yang JD, Gyedu A, Afihene MY (2015). Hepatocellular carcinoma occurs at an earlier age in African, particularly in association with chronic hepatitis B. Am J Gastroenterol.

[18] Kouandongui Bangue Songrou F, Kobelembi A, Djabanga C (2019). Diagnostic d’un carcinome hépatocellulaire à l’échographie à Bangui. J Afr Imag Med.

[19] Nsondé Malanda J, Diané S, Bolenga Liboko AF (2018). Diagnosis profile and therapeutic of hepatocellular carcinoma. SAJ Cancer Sci.

[20] Ranaivomanana M, Rakotovao A, Fanantenantsoa R (2017). Étude préliminaire épidémio-clinique des carcinomes hépatocellulaires observés au centre hospitalier Universitaire Tambohobe, Fianarantsoa. Rev Med Madag.

[21] Qari YA, Mosli MH (2017). Epidemiology and clinical feactures of patients with hepatocellular carcinoma at a tertiary hospital in Jeddah. Niger J Clin Pract.

[22] Bekondi C, Mobima T, Ouavène JO (2010). Étiopathologie du carcinome hépatocellulaire à Bangui, République centrafricaine: caractéristiques cliniques, biologiques et aspects virologiques des patients. Pathol Biol.

[23] Noah Noah D, Nko’Ayissi G, Ankouane Andoulo F (2014). Présentation clinique, biologique et facteurs de risque du carcinome hépatocellulaire: étude cas témoins à Yaoundé au Cameroun. Rev Med Pharm.

[24] Zakari N, Appolinaire S, Gilberte KC (2010). Carcinomes hépatocellulaires en milieu africain burkinabè: contribution de l’échographie à propos de 58 cas. Pan Afr Med J.

[25] Harir N, Zeggai S, Tou A (2016). Carcinome hépato-cellulaire dans l’Ouest Algérien: profil épidémiologiques et clinico-pathologiques. Rev Med Madag.

[26] Shankaran V, Chennupati S, Sanchez H (2021). Clinical characteristics, treatment patterns, and healthcare costs and utilization for hepatocellular carcinoma (HCC) patients treated at a large referral center in Washington state 2007-2018. J Hepatocell Carcinoma.

[27] Lee SS, Jeong SH, Byoun YS (2013). Clinical features and outcome of cryptogenic hepatocellular carcinoma compared to those of viral and alcoholic hepatocellular carcinoma. BMC Cancer.

[28] Zhao H, Zhu P, Han T (2020). Clinical characteristics analysis of 1180 patients with hepatocellular carcinoma secondary to hepatitis B, hepatitis C and alcoholic liver disease. J Clin Lab Anal.

[29] Huo TI, Ho SY, Liu PH (2021). Changing patterns of etiology and management of hepatocellular carcinoma: need for global reappraisal. J Gastroenterol.

[30] Ho SY, Liu PH, Hsu CY (2020). Evolution of etiology, presentation, management and prognostic tool in hepatocellular carcinoma. Sci Rep.

[31] Enomoto H, Ueno Y, Hiasa Y (2021). The transition in the etiologies of hepatocellular carcinoma complicated liver cirrhosis in a nationwide survey of Japan. J Gastroenterol.

[32] Lim MS, Goh GBB, Chang JPE (2021). A study of 3013 cases of hepatocellular carcinoma and therapy before and during the current decade. JGH Open.

[33] Wands J (2007). Hepatocellular carcinoma and sex. N Engl J Med.

[34] Johnson JP, Leung N, Cheng P (1997). ‘Hepatoma-specific’ alpha-fetoprotein may permit preclinical diagnosis of malignant change in patients with chronic liver disease. British J Cancer.

[35] Forner A, Reig M, Bruix J (2009). Alpha-fetoprotein for hepatocellular carcinoma diagnosis: the demise of a brilliant star. Gastroenterology.

[36] Appel-da-Silva MC, Miozzo SAS, Dossin IA (2016). Incidence of hepatocellular carcinoma in outpatients with cirrhosis in Brazil: a 10-year retrospective cohort study. World J Gastroenterol.

[37] Zeller HG, Rakotonirina J, Morel B (1996). Etiologie des hépatocarcinomes à Madagascar: Résultats d’une étude menée à Antananarivo d’octobre 1995 à Octobre 1996. Arch Inst Pasteur Madagascar.

[38] Chuang SC, Lee YC, Hashibe M (2010). Interaction between cigarette smoking and hepatitis B and C virus infection on the risk of liver cancer: a meta-analysis. Cancer Epidemiol Biomarkers Prev.

[39] Gong Y, Egal S, Hounsa A (2003). Determinants of aflatoxin exposure in young children from Benin and Togo, West Africa: the critical role of weaning. Int J Epidemiol.

[40] El-Zayadi AR, Badran HM, Barakat EMF (2005). Hepatocellular carcinoma in Egypt: a single center study over a decade. World J Gastroenterol.

